# Successful ECMO treatment in patients with cerebral hemorrhage and PROC gene mutation associated with VTE: a case report

**DOI:** 10.1186/s12959-024-00601-y

**Published:** 2024-04-12

**Authors:** Lijie Wang, Chengyong Ma, Luping Wang, Qianrong Ding, Hao Yang, Bo Wang, Qin Wu

**Affiliations:** Department of Critical Care Medicine, West China Hospital of Sichuan University, Sichuan Province, No. 37, Guo Xue Xiang, Chengdu, China

**Keywords:** ECMO, Anticoagulation, PROC, VTE, Pulmonary Embolism

## Abstract

In this report, we report a case of a middle-aged male, admitted to the ICU with cerebral hemorrhage resulting from a severe high-altitude fall. The patient encountered significant challenges in oxygenation index correction, attributed to extensive embolism in both the primary and branch pulmonary arteries. Consequently, the patient underwent an immediate initiation of veno-arterial extracorporeal membrane oxygenation (VA-ECMO) therapy, persisting for 20 days. During this treatment period, a mutation in the protein C (PROC) gene was identified. The medical team meticulously navigated the delicate balance between anticoagulation and bleeding risks. Eventually, the patient was successfully weaned off VA-ECMO and subsequently discharged. This report aims to delve into the etiology and therapeutic approaches of this uncommon case, with the intention of offering insightful reference for managing similar clinical scenarios in the future.

**Supplementary Information:**

The online version contains supplementary material available at 10.1186/s12959-024-00601-y.

## Background

PROC, a vitamin K-dependent anticoagulant protein synthesized in the liver, is composed of two chains connected by disulfide bonds [1]. It circulates as a zymogen and is activated into activated protein C (aPC), which exerts an anticoagulant effect primarily by inactivating coagulation factors V and VIII [2]. PROC deficiency is classified into inherited and acquired types. To date, over 160 distinct mutations in the PROC have been identified [3, 4], with the gene located on chromosome 2 (2q13-14) [5, 6]. Individuals with PROC deficiency are susceptible to venous thromboembolism (VTE) at various sites, with the most frequent manifestations being deep vein thrombosis (DVT) of the lower extremities, mesenteric vein thrombosis, and pulmonary embolism (PE) [7, 8]. This report highlights a case where a definitive diagnosis of PROC deficiency was made in a patient who, despite suffering from cerebral hemorrhage and VTE, was successfully treated using extracorporeal membrane oxygenation (ECMO) therapy.

## Case presentation

The patient, following a fall from approximately 2 m, was admitted with a head injury, manifesting as disturbances in consciousness and bleeding from the left external auditory canal. Within an hour of admission, the patient exhibited limb convulsions and further disturbances in consciousness. Computed tomography (CT) scan of the head revealed subdural, epidural, and subarachnoid hemorrhages. Management included administration of tranexamic acid for hemostasis and sodium valproate for seizure control. On 12th May, 2023, the patient developed a high fever and significant hypoxemia. Computed tomography pulmonary angiography (CTPA) revealed extensive embolism in the main and branch pulmonary arteries, with the main trunk measuring 4.3 cm in width. After initial ECMO treatment, the patient was transferred to our hospital. On admission, clinical examination showed a temperature of 36.5 °C, heart rate of 90 beats per minute, blood pressure of 130/80 mmHg, and the patient was under sedation and analgesia, with tracheal intubation connected to a ventilator.

**Fig. 1:**
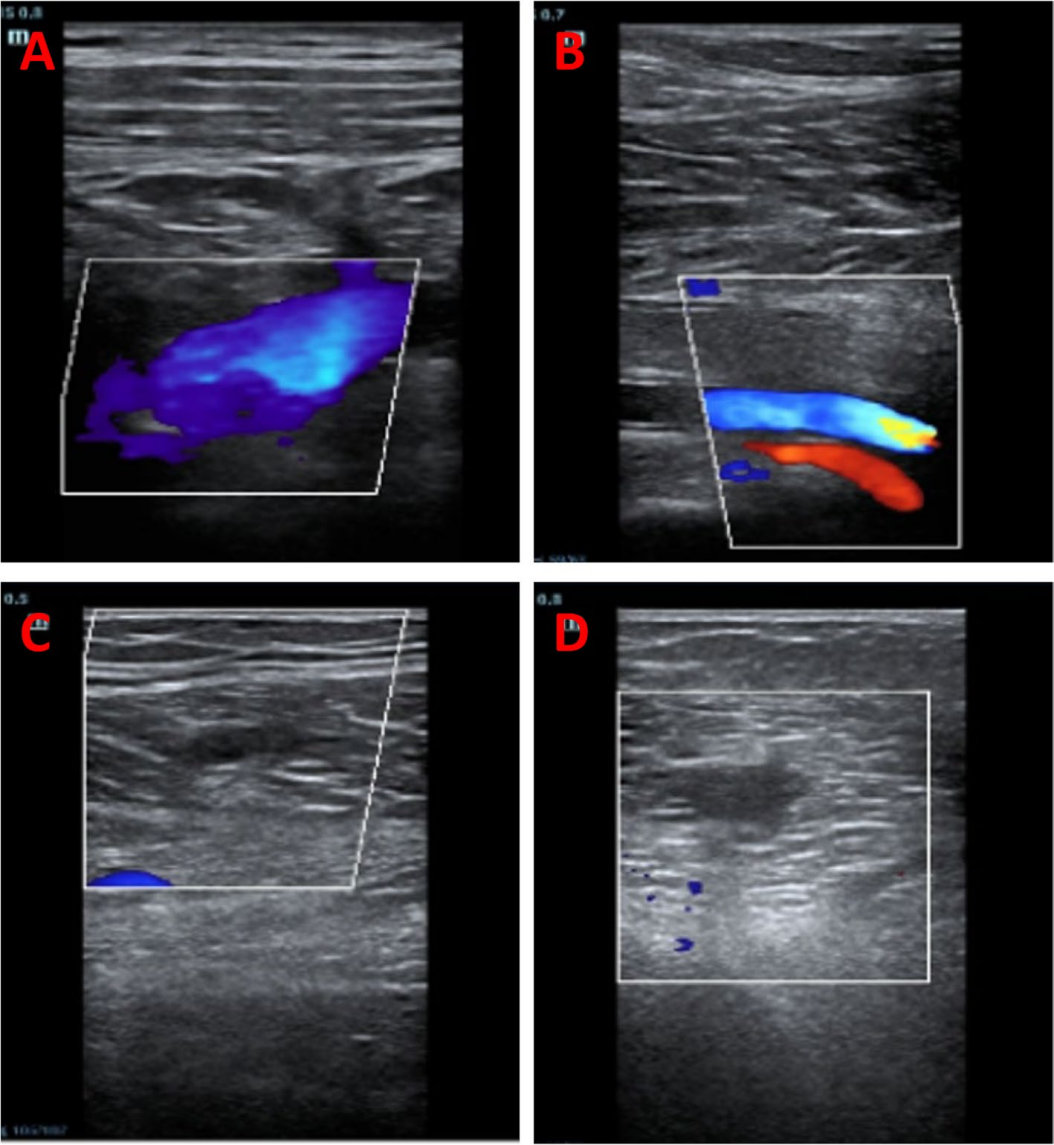
Ultrasound of limbs and internal jugular vein on 14th May. **A** left subclavian vein; **B** left popliteal vein; **C** left intermuscular calf vein; **D** Right intermuscular calf vein

**Fig. 2:**
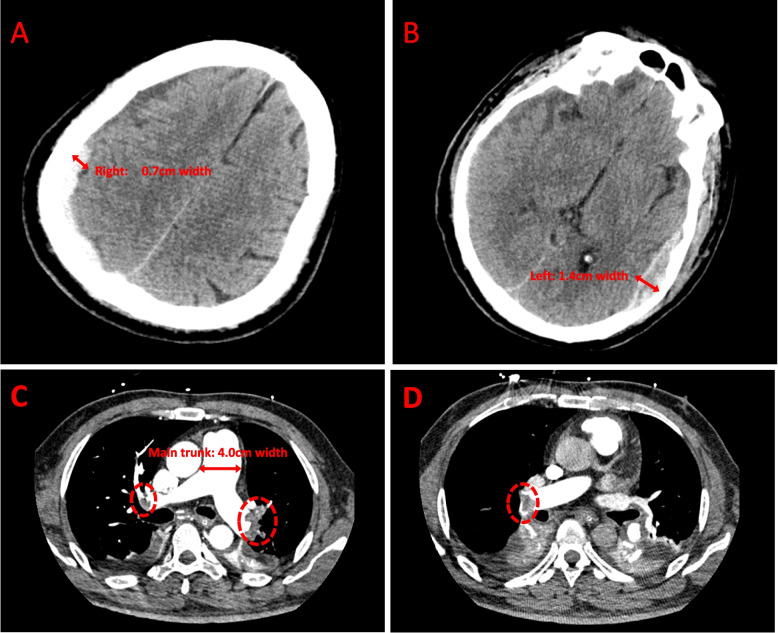
Head CT and CTPA on 15th May. **A** A crescent-shaped high-density shadow under the inner plate of the right top skull, indicating a right subdural hematoma, the widest part of which is about 0.7cm; **B** The left temporal fusiform mixed high-density shadow, indicating the left epidural hematoma, the widest hematoma about 1.4cm; **C** and **D** show pulmonary arteries embolized at different levels of the lungs, Pulmonary artery thickening, the maximum width of the main trunk is about 4.0cm, the left and right pulmonary trunk distal and branches filled with multiple defects, suggesting pulmonary embolism. (Note: The places marked in the circle are thrombus of the pulmonary artery)

**Fig. 3:**
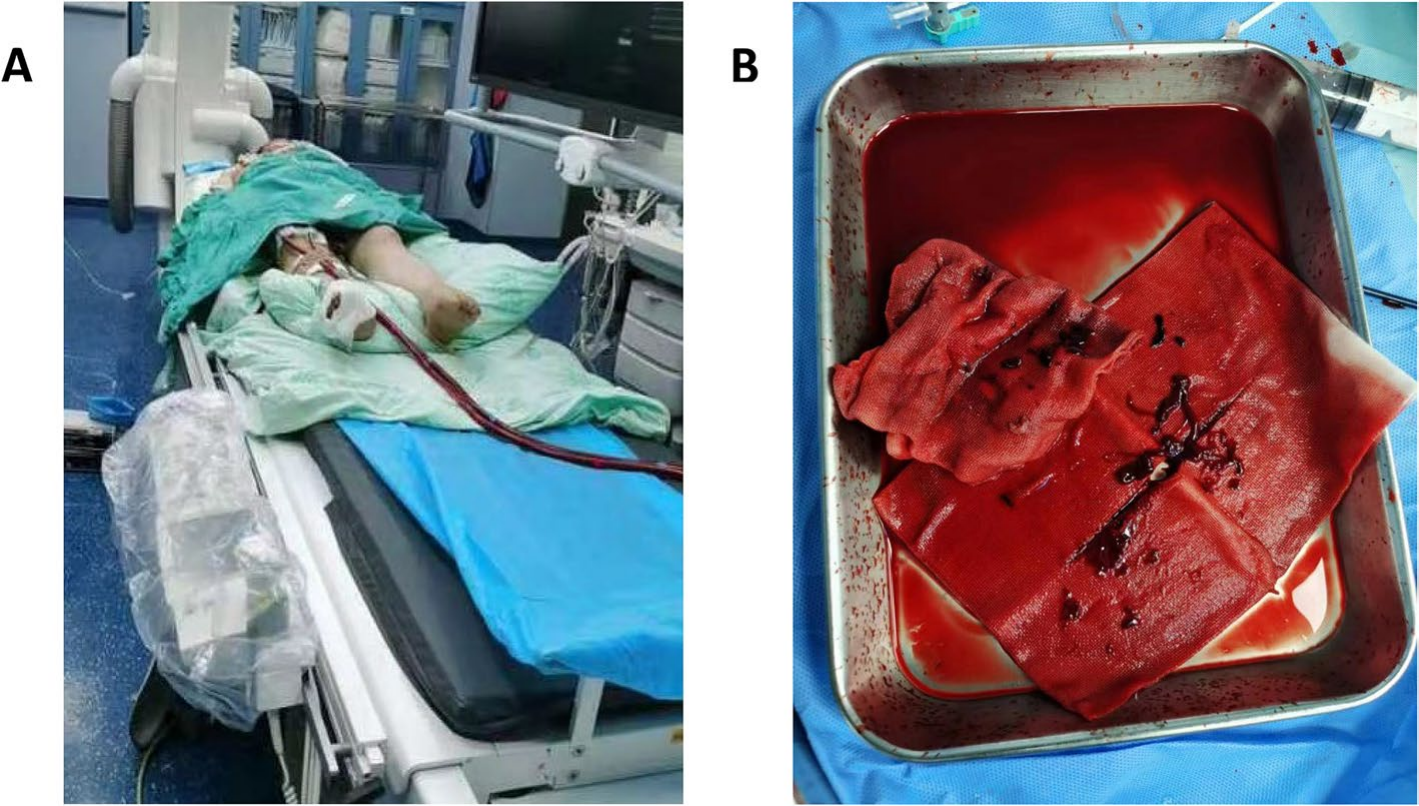
Interventional pulmonary angiography and pulmonary embolization on 17th May. **A** Patients are sent to the operating room during ECMO treatment. **B** Vascular surgery performed pulmonary angiography with interventional catheter and pulmonary thrombus on 17th May

CT scans indicated subdural and epidural hematomas on 13th May 2023 (Supplementary Fig. 1). Continuous ECMO therapy was administered from 13 to 15th May, with heparin dosing modulated based on the patient’s activated partial thromboplastin time (aPTT) and activated clotting time (ACT) (refer to Table 1). Ultrasound findings on 14th May displayed conditions of the limbs and internal jugular vein (Fig. 1). On 15th May, CTPA showed pulmonary artery thickening and multiple embolisms (Fig. 2). After a multidisciplinary consultation, interventional pulmonary angiography and embolization were performed on 17th May (Fig. 3). Postoperatively, the patient’s oxygenation index improved significantly, necessitating an increase in heparin to 1400IU/h (Table 1). Repeated imaging on 18th May (Supplementary Figs. 2 and 4) and 22th May (Supplementary Fig. 3) revealed recurrent thrombosis. Following another multidisciplinary discussion on 23th May, local thrombolysis was recommended due to the patient’s history of cerebral hemorrhage. On 24th May, catheter intervention for lower pulmonary artery thrombolysis and inferior vena cava filter implantation were performed (Supplementary Fig. 5A). Between 25 and 26th May, anticoagulation therapy with heparin and rivaroxaban was administered without substantial improvement in oxygenation. Bivalirudin therapy was initiated on 26th May (Table 1). On 27th May, severe bleeding at the ECMO puncture site necessitated the cessation of thrombolytic therapy (Supplementary Fig. 5B, C), and bivalirudin anticoagulation was continued. Oxygenation and coagulation parameters stabilized by 1st June, allowing for the successful withdrawal of ECMO.

**Table 1:** Coagulation function, anticoagulation regimen and ECMO parameters of patients in different periods

On ECMO days		1	2	3	4	5	6	7	8	9	10	11	12	13	14	15	16	17	18	19	20
Date		13	14	15	16	17	18	19	20	21	22	23	24	25	26	27	28	29	30	31	1
Month		May																			June
Arterial blood gases (ABG)	PH	7.422	7.426	7.394	7.395	7.406	7.398	7.429	7.398	7.374	7.409	7.403	7.412	7.411	7.424	7.43	7.443	7.446	7.444	7.462	7.463
	PO2	52.4	64.1	71.8	80.6	129.9	89.7	91.7	81	103.1	117.4	102	107.8	102.5	102.4	85.4	99.6	123.9	138.8	125.7	135.4
	PCO2	40.4	43.6	47	45.5	42.7	43.4	40.2	47	48.8	44.6	43.9	42	42.2	43.7	44.9	46.7	41.5	41.6	42.5	42.1
	Lac	2.2	1.3	1.1	< 1.0													1.1		1.3	1.2
Ventilator parameters	Ventilator mode	A/C(VC)																			
	VT(mL)	450																			
	FiO2	100%			60%		70%		60%							100%	90%	80%	40%		
	PEEP(cmH2O)	8			10													12	14		
Oxygenation index		52.4	64.1	71.8	80.6	162.4	179.4	183.4	162	206.2	234.8	255	215.6	256.35	256	85.4	124.5	123.9	462.7	419	451.3
ECMO parameter	RPM	3380	3410	3660	3551	4000	4000	3480	3575	3575	3575	3700	3702	3705	3575	3895	3895	3170	3535	2786	3010
	LPM(L/min)	4.22	3.56	3.7	3.82	3.98	4.12	3.55	3.78	3.78	3.8	3.94	3.89	3.81	3.75	4.24	4.13	3.33	3.18	2.29	2.16
	FiO2	100%				80%	50%							40%		100%	80%	100%	30%		
	Gas flow(L/min)	2								3											
	Anticoagulation (Heparin-IU/h or Bivaludin-mg/kg/h)	Heparin 0–800	Heparin 1000–1200	Heparin 1000				Heparin 1000–1200	Heparin 1200	Heparin 1200–1400	Heparin 1400				Bivaludin 0.1–0.2	Bivaludin 0.09–0.1	Bivaludin 0.09		Bivaludinn 0.07–0.08	Bivaludin 0.07	
Coagulation function	APTT	28.08	27.77	29.48	30.48	27.34	28.2	30.2	32.2	31.4	32.47	31.94	36.9	31.95	33.76	43.78	43.8	41.17	42.42	38.98	37.73
	ACT	159.9	157.15	168.3	167.9	165.83	174.17	173	179.33	174.67	170.58	171.5	179.45	186.64	200.23	212.71	214.64	216.5	219	213.5	211
	Thrombin III	78.9	81.9	82.1	89.2	92	92.7	92.4	97.5	93.9	87.7		100.1	93.1	100.4	98.82	98.57	102.92	97.72	104.73	107.63
	D-dimer	> 38																	33.5	22.87	23.96
Income and output	Income (ml)	1380	2475	2218.4	1998.4	2448.4	3071.8	2300	2965	3216.8	2510	2450	2702	2230	2890	2585	2350	2842	2646	2678	2219
	Output (ml)	2030	2674	3398	2979	3097	2244	2748	3726	3057	2337	3317	3131	2784	3099	2434	2039	1919	2906	3274	1852
SOFA		11	9	9	11	7	8	7	8	6		7

Warfarin therapy commenced on 5th June, with a gradual reduction in bivalirudin. The warfarin dosage was fine-tuned from 14th June based on the patient’s international normalized ratio (INR) levels (Supplementary Fig. 6). Due to the patient’s heavy thrombus load during treatment and insensitivity to heparin therapy, we conducted testing for 192 thrombosis and hemostatic genes on 25th June. The results revealed two point mutations in the PROC gene (PROC:NM_000312:exon7:c. 572_574del:p.K193del and PROC:NM_000312:exon8:c.A703C:p.K235Q) and no mutations in the CYP2C9 and VKORC1 genes (Supplementary Table 1). The patient was discharged in a stable condition on 18th July.

After discharge, the patient was instructed to regularly monitor INR, with warfarin dosages adjusted under professional guidance. A CTPA conducted on 25th October indicated a reduction in pulmonary artery filling defects (Supplementary Fig. 7).

## Discussion

PROC is activated on the endothelial cell membrane, necessitating two membrane receptors: endothelial PROC receptor and thrombomodulin [9, 10]. aPC mitigates thrombin generation by selectively proteolyzing activated factors V and VIIIa [1, 11]. While severe PROC deficiency (homozygous or compound heterozygous forms) is exceedingly rare (prevalence between 1/500,000 and 1/750,000), partial deficiencies (heterozygous forms) are relatively common (occurring in 1/200 to 1/500 individuals) [11, 12]. In our observed cases, patients exhibited two point mutations in the PROC gene (refer to Supplementary Table 1). Notably, despite the administration of large heparin doses for anticoagulation in patients with DVT, resistance to heparin was suspected. This suspicion prompted a switch to alternative anticoagulants, leading to a marked reduction in systemic blood clots and a decrease in reliance on ECMO and ventilatory support parameters (Fig. 1). Heparin resistance typically involves the need for unusually high heparin doses to maintain therapeutic aPTT (or ACT) levels, often due to enhanced heparin clearance or elevated factor VIII levels [13, 14]. In this context, PROC deficiency contributes to factor VIII elevation, potentially leading to heparin resistance. Consequently, we propose comprehensive thrombogenic screening in young patients presenting with unexplained multiple VTE. If new VTEs emerge despite high-dose anticoagulant therapy, consideration of heparin resistance should prompt a swift switch in anticoagulant medication.

A repeat CT of the head on 15th May, three days post-admission, revealed a progression in the extent of intracranial hemorrhage (Fig. 2). Despite this, continued ECMO was necessary, warranting anticoagulation to ensure circuit patency. Managing anticoagulation in this scenario posed a significant challenge. For veno-venous extracorporeal membrane oxygenation (VV-ECMO), standard practice involves administering a heparin loading dose (e.g., 5000U) prior to intubation, followed by a continuous intravenous infusion, with the aim of maintaining aPTT within 40–60 s. However, this anticoagulation protocol may be adjusted in cases with additional factors necessitating higher anticoagulation levels, such as venous thromboembolism, atrial fibrillation, or thrombosis, or if anticoagulation is contraindicated due to bleeding or procedural requirements [15, 16].

Studies have explored low-intensity anticoagulation in traumatic brain injury (TBI) patients, maintaining aPTT between 45 and 55 s without exacerbating intracranial hemorrhage [17]. In one study focusing on TBI patients undergoing VV-ECMO, a maintained aPTT between 45 and 55 s showed that among 29 patients (81%) undergoing repeat head CT during ECMO, only one exhibited hematoma enlargement and another developed a new bleeding site [18]. Conversely, in VA-ECMO cases without active bleeding, a higher aPTT target is often pursued due to increased risks of arterial embolism, left ventricular thrombosis, and circuit thrombosis, which are associated with arterial cannulation, retrograde arterial reinfusion, and lower blood flow rates compared to VV-ECMO. Thus, an aPTT range of 45 to 55 s is deemed safe for patients with cerebral hemorrhage. However, in our patient’s case, the target aPTT was not achieved during ECMO therapy, primarily due to a PROC gene defect leading to a degree of heparin resistance.

Currently, the management of severe PROC deficiency primarily involves the use of PROC substitutes and oral anticoagulants. Exogenous PROC can be administered either through fresh frozen plasma or as a concentrated pharmaceutical preparation derived from purified PROC [19, 20]. Another critical aspect of long-term management in these patients is the administration of long-term oral anticoagulants, such as non-vitamin K antagonist oral anticoagulants (NOACs) [21]. The selection between NOACs and traditional anticoagulants like warfarin depends on various factors, including the severity of thrombosis, patient preference, and adherence to treatment. Research indicates that indefinite anticoagulation is necessary for patients with PROC deficiency, particularly those with a significant family history of VTE [22].

In the case of our patient, warfarin was chosen as the anticoagulant. We conducted regular monitoring of the INR, evaluated for extremity venous thrombosis, and performed CTPA. The dosage of warfarin was adjusted based on these results, ensuring effective and tailored anticoagulation management.

### Supplementary Information

**Supplementary Material 1.** 

## Data Availability

No datasets were generated or analysed during the current study.

## Abbreviations

VA-ECMO: Veno-arterial extracorporeal membrane oxygenation
ECMO: Extracorporeal membrane oxygenation
VV-ECMO: Veno-venous extracorporeal membrane oxygenation
PROC: Protein C
aPC: Activated protein C
VTE: Venous thromboembolism
DVT: Deep vein thrombosis
PE: Pulmonary embolism
CT: Computed tomography
CTPA: Computed tomography pulmonary angiography
aPTT: Activated partial thromboplastin time
ACT: Activated clotting time
INR: International normalized ratio
TBI: Traumatic brain injury
NOACs: Non-vitamin K antagonist oral anticoagulants
