# Supplementary material for: Successful ECMO treatment in patients with cerebral hemorrhage and PROC gene mutation associated with VTE: a case report
Source: Thromb J. 2024 Apr 12;22:36. doi: 10.1186/s12959-024-00601-y (PMC11010424; doi:10.1186/s12959-024-00601-y)
Supplement: Supplementary file 1 — Supplementary Material 1. [file 12959_2024_601_MOESM1_ESM.docx]

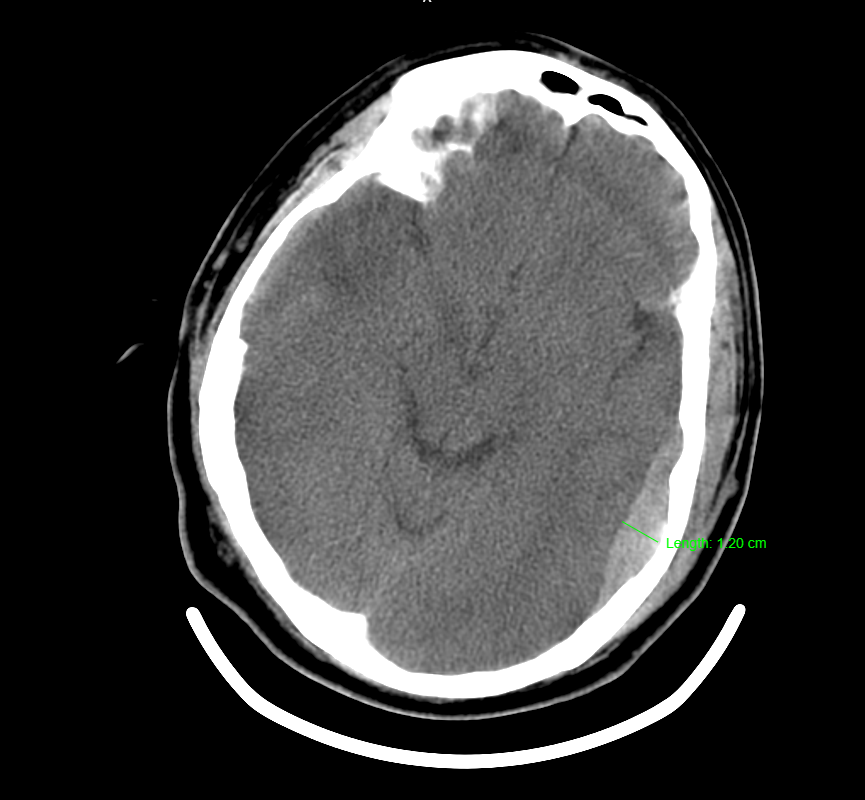

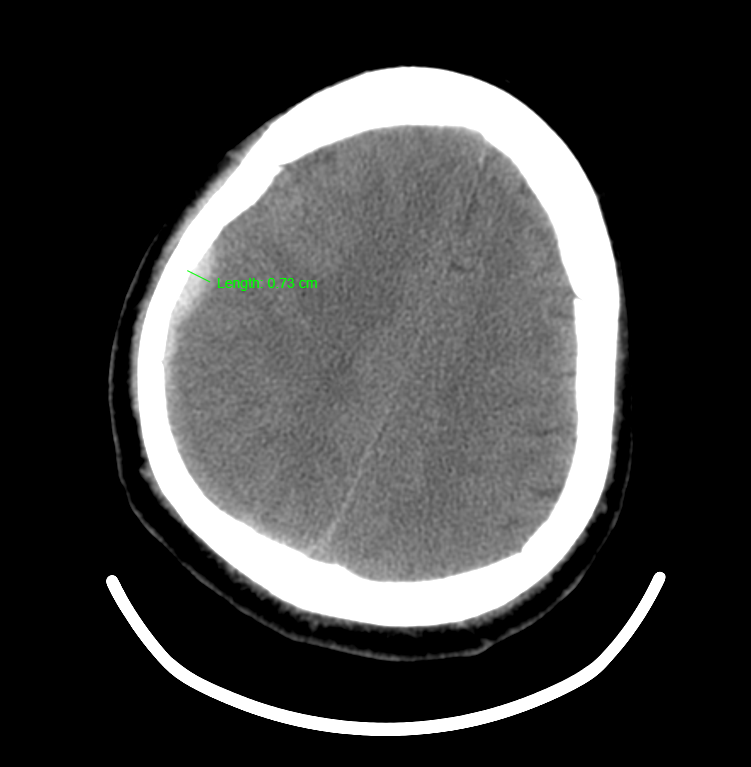


**Supplementary Figure 1. Head CT on 13^th^ May.**

A slightly high-density shadow was observed under the medial parietotemporal plate on both sides, with a width of 0.7cm on the right side and 1.2cm on the left side, and subdural or extra-dural hematoma

A

B


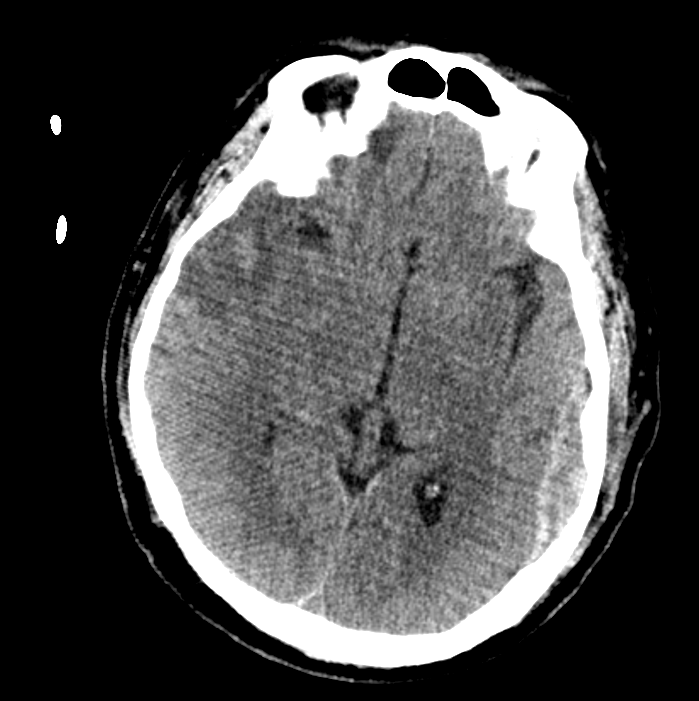

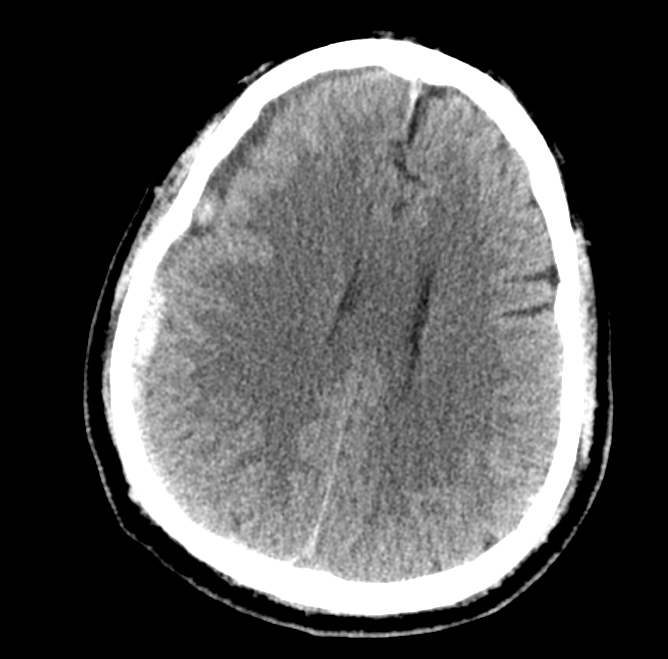

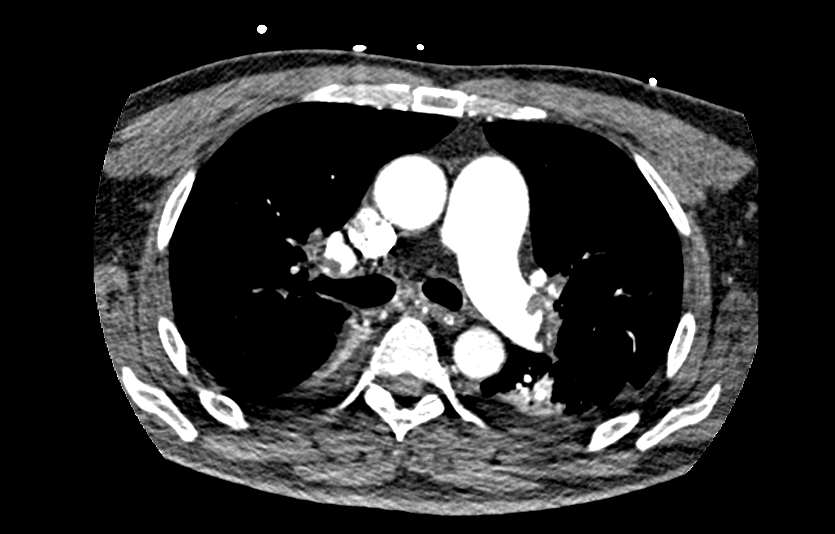

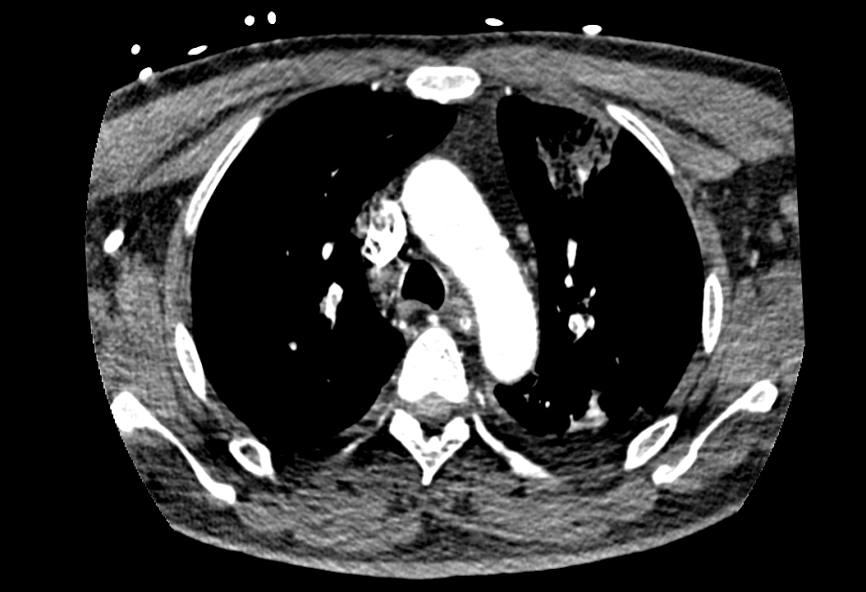


**Supplementary Figure 2. Head CT and CTPA on 18^th^ May.**

(A slightly high-density shadow was observed under the medial parietotemporal plate on both sides, with a width of 0.7cm on the right side and 1.4cm on the left side, and subdural or extra-dural hematoma. Pulmonary artery thickening, the maximum width of the main trunk is about 4.0cm, the left and right pulmonary artery trunk distal and branches filled with multiple defects, suggesting pulmonary embolism.)

**C**

**A**

**B**

**D**


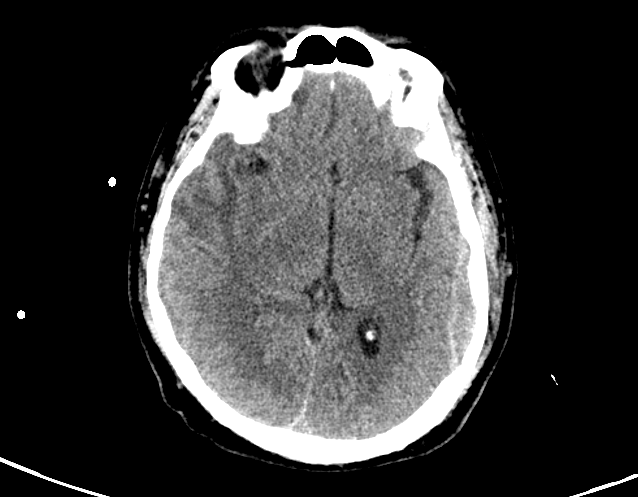

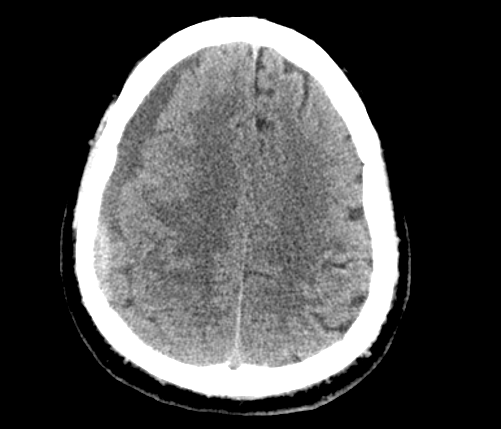

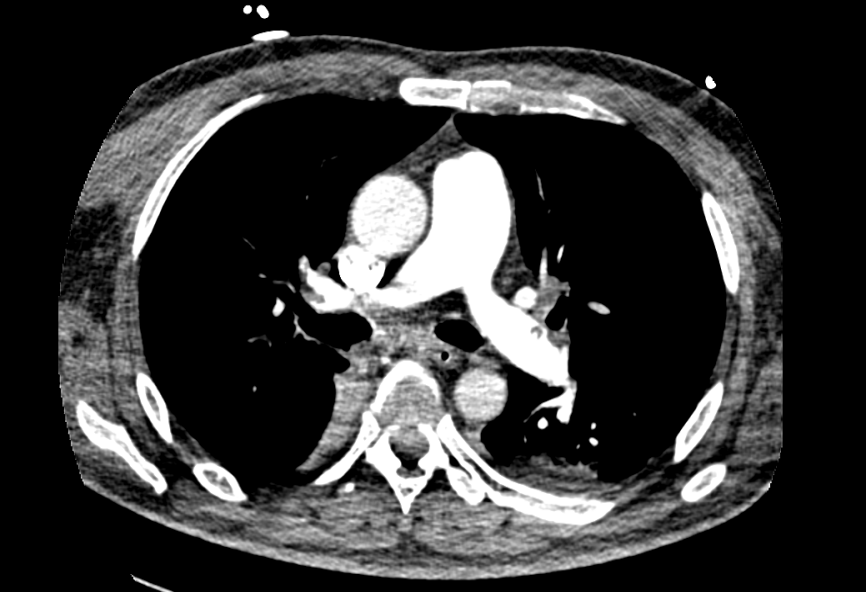

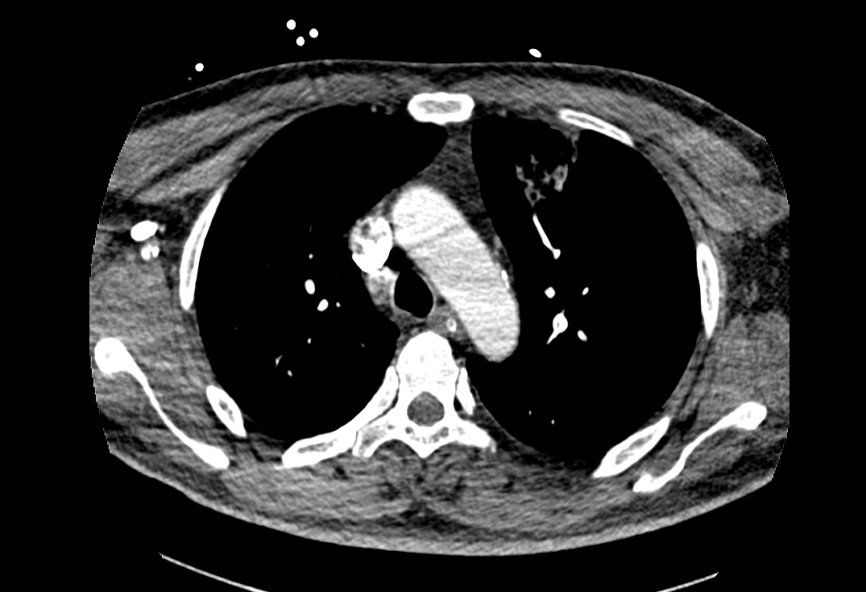


**Supplementary Figure 3. Head CT and CTPA on 22^th^ May.**

(A slightly high-density shadow was observed under the medial parietotemporal plate on both sides, with a width of 0.6cm on the right side and 1.2cm on the left side, and subdural or extra-dural hematoma. Pulmonary artery thickening, the maximum width of the main trunk about 3.9cm, left and right pulmonary trunk distal and branches filled with multiple defects, suggesting pulmonary embolism.)

**A**

**B**

**C**

**D**


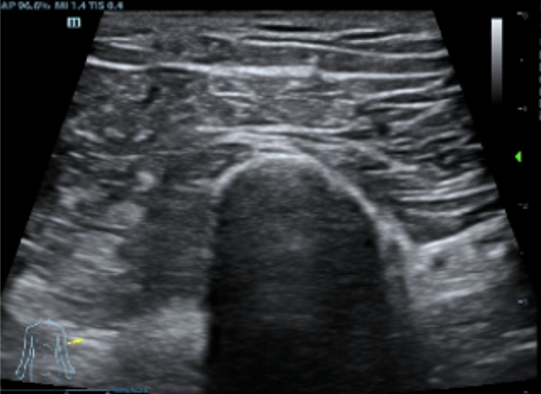

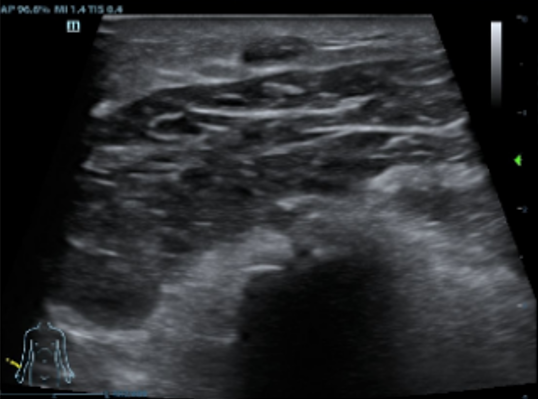

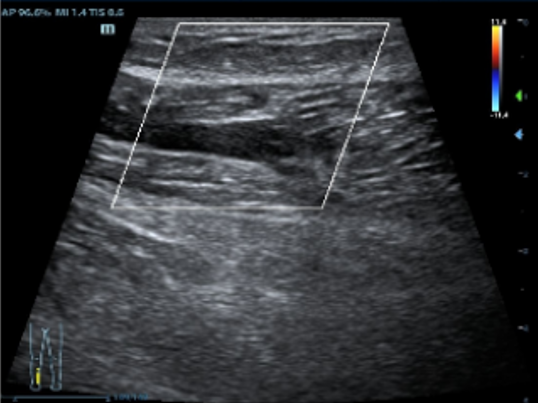

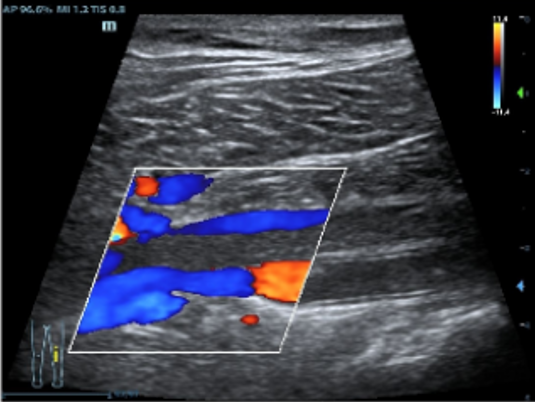


**Supplementary Figure 4. Venous ultrasound of the extremities on 18^th^ May.**

(Bilateral cephalic vein thrombosis, left popliteal vein and bilateral partial calf intermuscular vein thrombosis. (A) right intermuscular vein of lower leg; (B) left popliteal vein; (C) Forearm segment of right cephalic vein; (D) Upper brachial segment of left cephalic vein.)

**C**

**A**

**B**

**D**


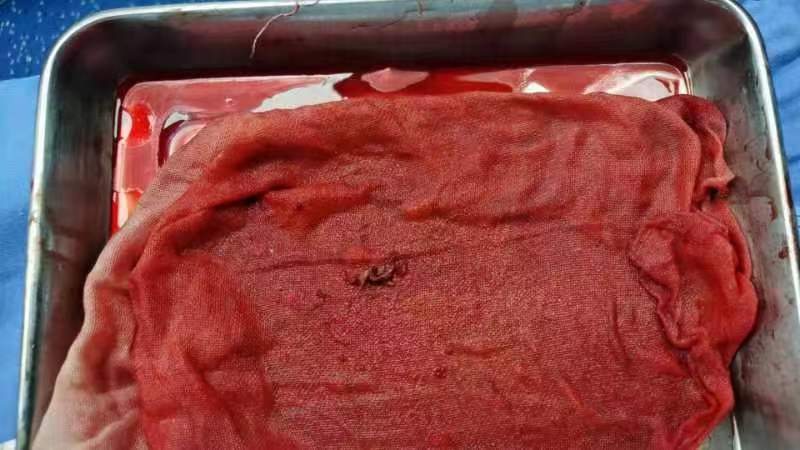

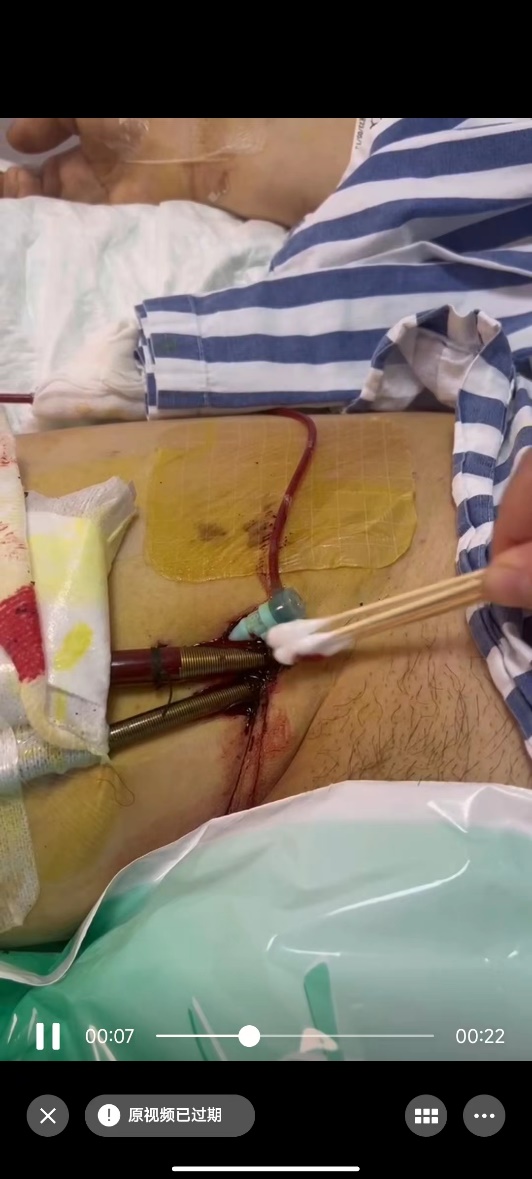

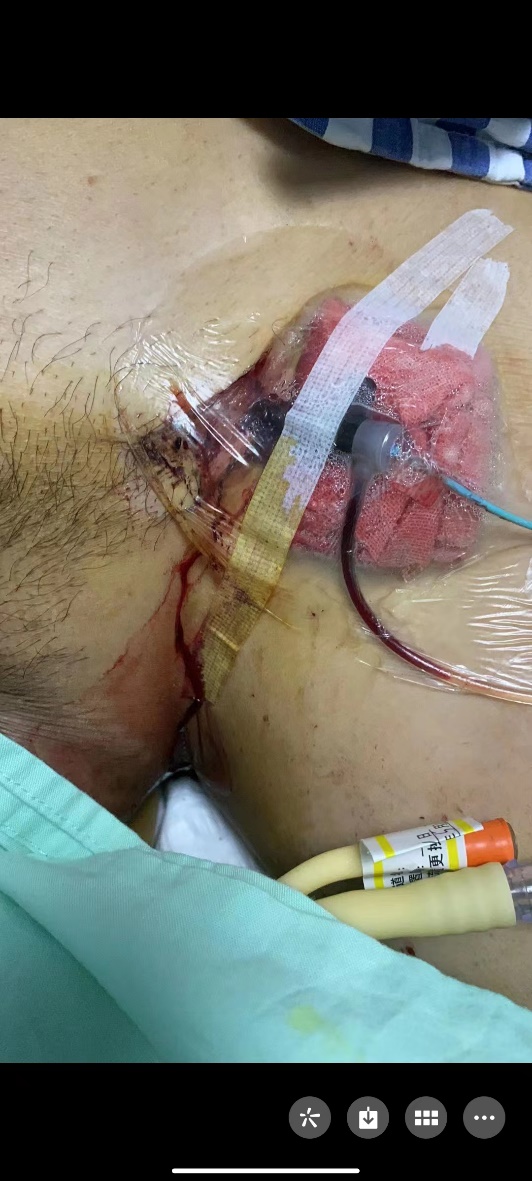


**Supplementary Figure 5. The second interventional surgery and postoperative surgery.**

(A The thrombus extracted from the patient at the time of the second suction thrombus operation 24th May; B and C extravasation of blood from the ECMO puncture site after thrombolysis.)

**A**

**B**

**C**


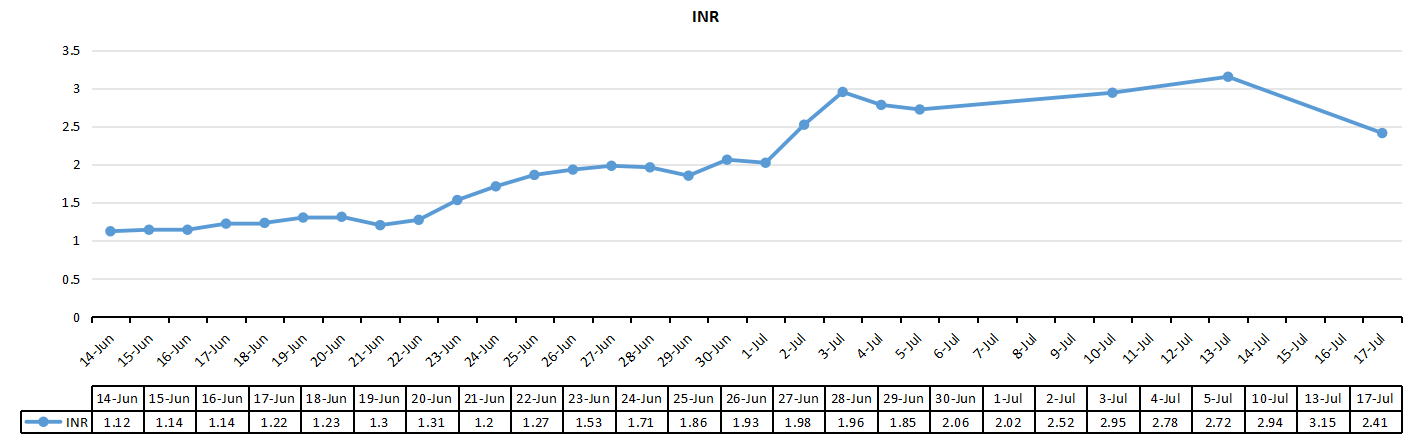

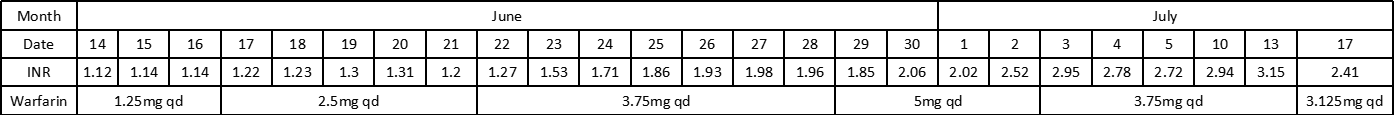


**Supplementary Figure 6. The dosage of warfarin was adjusted according to the patient's INR level.**


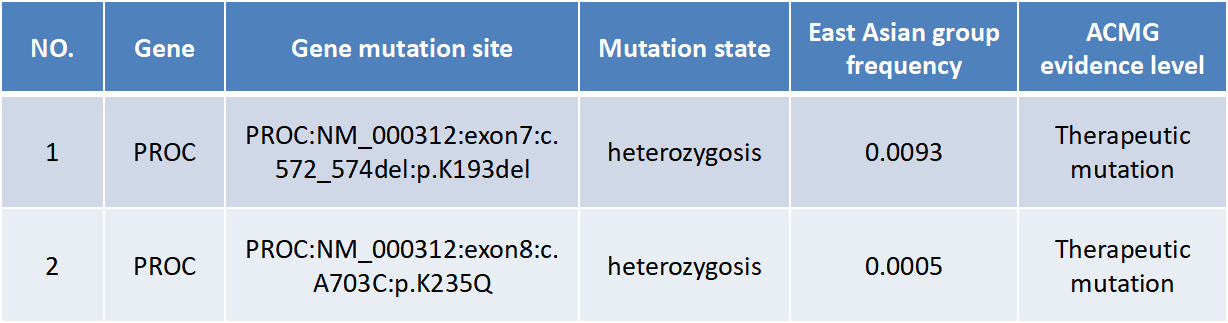


**192 thrombosis and hemostatic genes including:** A2M, ABCB1, ABCG2, ABCG5, ABCG8, ABO, ACE, ACTN1, ADAMTS13, ADGRB3, ADTRP, ANKRD26, ANO6, ANXA5, AP3B1, AP3D1, APOH, APOM, ARPC1B, ATOH1, B3GAT2, BAZ1B, BCOR, BCORL1, BDKRB2, BLOC1S3, BLOC1S6, BLZF1, C4BPA, C4BPB, CADM1, CALR, CBS, CES1, CETP, CFH, CHST14, COL1A1, COL28A1, COL3A1, COL5A1, COL5A2, CPB2, CYCS, CYP2C19, ***CYP2C9***, CYP3A4, CYP4F2, CYP4V2, DIAPH1, DTNBP1, EDEM2, EDN1, ETV6, F10, F11, F12, F13A1, F13B, F2, F3, F5, F7, F8, F9, FERMT3, FGA, FGB, FGG, FLI1, FLNA, FTO, FYB1, GATA1, GCKR, GFI1B, GGCX, GNE, GP1BA, GP1BB, GP6, GP9, HABP2, HBA1, HBA2, HBB, HBD, HBE, HBG1, HBG2, HFE, HIVEP1, HMOX1, HOXA11, HPS1, HPS3, HPS4, HPS5, HPS6, HRG, IGFBP3, IL12RB1, IL18, IL1RL1, IL6, ITGA2B, ITGB3, JAK1, JAK2, JAK3, KDSR, KLKB1, KNG1, LEMD3, LMAN1, LOC100130298, LPA, LY86, LYST, MCFD2, MECOM, MPIG6B, MPL, MRPL37, MTHFR, MTR, MTRR, MYH9, NAT8B, NBEA, NBEAL2, NME7, NOS3, NR1I2, ORAI1, P2RY12, PEAR1, PIGA, PLA2G4A, PLAT, PLAU, PLG, PRKN, ***PROC***, PROCR, PROS1, PROS2P, PROZ, PTGS2, PTPN11, RAC1, RASGRP2, RBM8A, RGS7, RIMS1, RNU4ATAC, RUNX1, SELE, SELP, SERPINA1, SERPINA10, SERPINA5, SERPINC1, SERPIND1, SERPINE1, SERPINF2, SH2B3, SLC44A2, SLC4A1, SLFN14, SMAP1, STAB2, STIM1, STXBP2, STXBP5, TBXA2R, TBXAS1, TC2N, TFPI, THBD, THPO, THSD7A, TLR9, TPM4, TSPAN15, TUBB1, VIPAS39, ***VKORC1***, VPS33B, VWF, WAS, ZFPM2

**Supplementary Table 1. Thrombus and hemostatic gene Panel detection report**


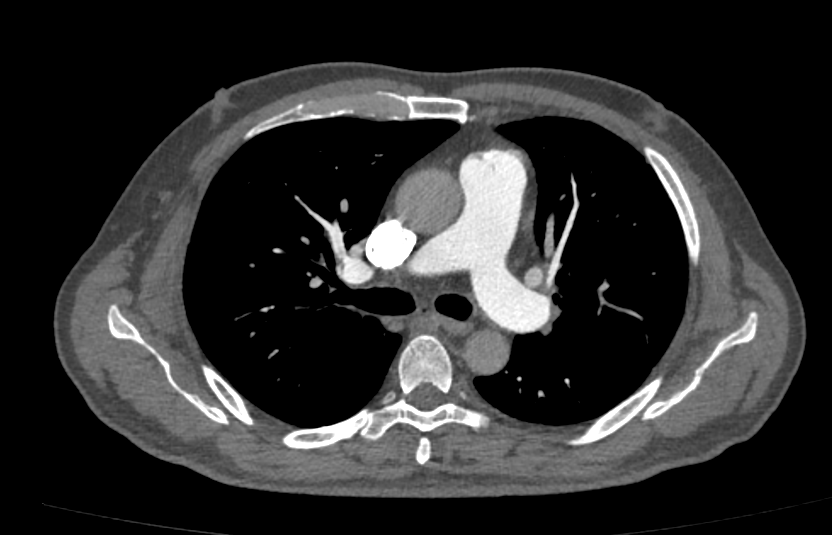

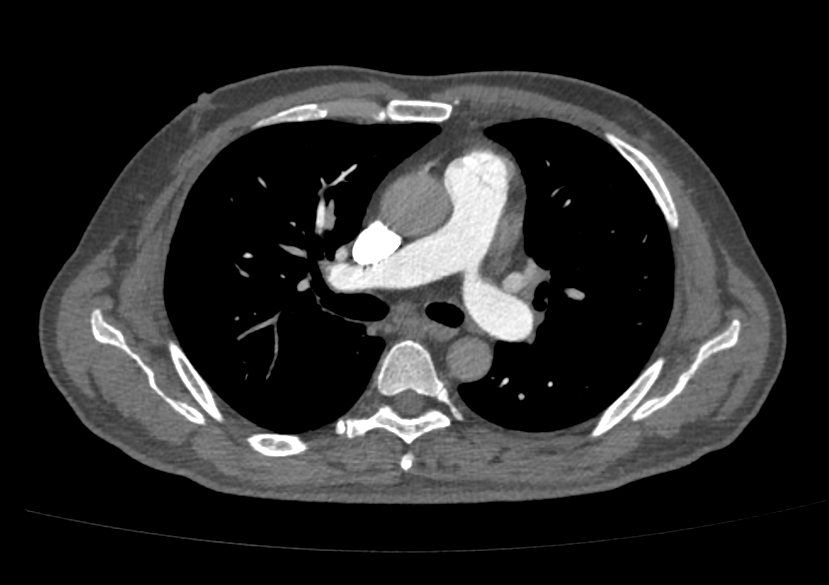

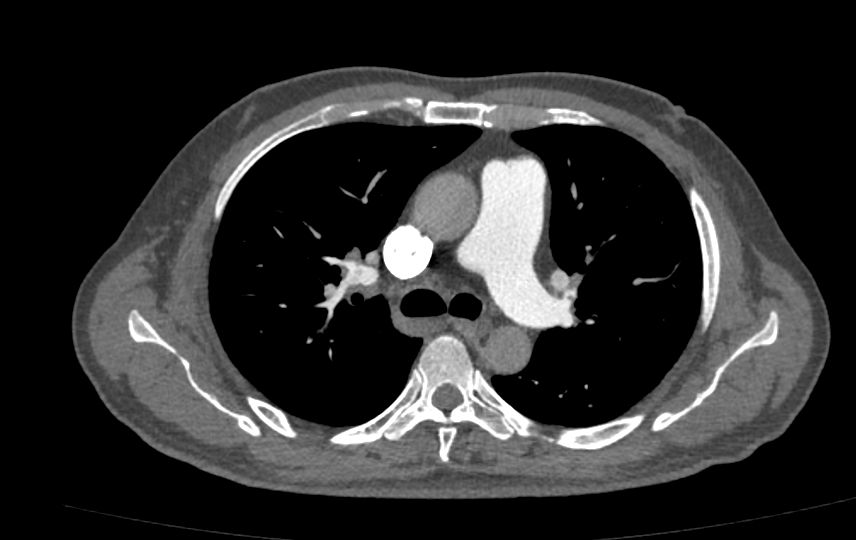

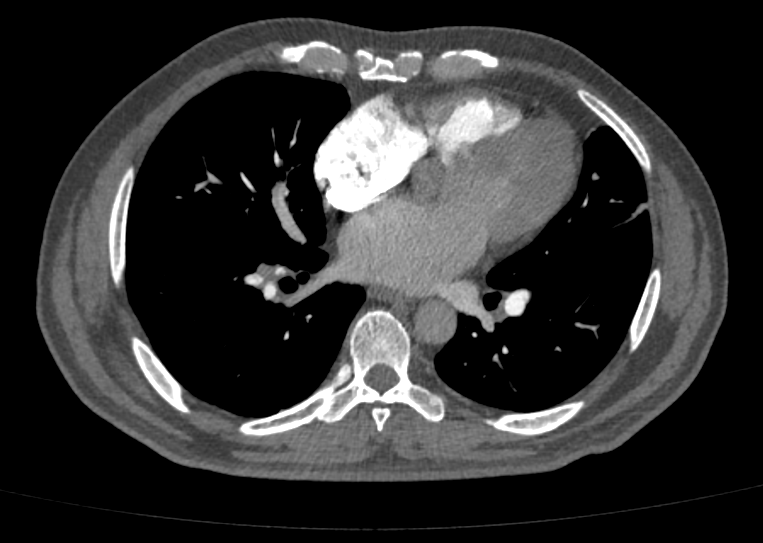


**A**

**B**

**C**

**D**

**Supplementary Figure 7. CTPA was reviewed 3 months after discharge (25^th^ October 2023).**

(Compared with CT on 19^th^ June 2023, the filling defect of the right pulmonary trunk and left and right pulmonary artery branches was reduced)
